# Prokaryotic Pangenomes Act as Evolving Ecosystems

**DOI:** 10.1093/molbev/msac232

**Published:** 2022-10-27

**Authors:** James O McInerney

**Affiliations:** School of Life Sciences, The University of Nottingham, University Park, Nottingham NG7 2UH, UK

**Keywords:** pangenomes, prokaryote, ecosystem

## Abstract

Understanding adaptation to the local environment is a central tenet and a major focus of evolutionary biology. But this is only part of the adaptionist story. In addition to the external environment, one of the main drivers of genome composition is genetic background. In this perspective, I argue that there is a growing body of evidence that intra-genomic selective pressures play a significant part in the composition of prokaryotic genomes and play a significant role in the origin, maintenance and structuring of prokaryotic pangenomes.

Sometimes in science, experiments are inspired by the ideas that precede them. At other times, unexpected data comes along first, and the task is to develop the theory that explains the observed patterns. Twenty years ago, three *Escherichia coli* genomes became available (Welch et al. 2002) and provided us with the first insights into how much variation might be seen in the genomes of a single prokaryotic species. Journals like PNAS discourage the use of superlatives in the text of a paper, but on this occasion, the abstract contained the word “amazingly”, simply because it was probably the best word to describe the finding. What was amazing was that only 39.2% of the combined, nonredundant, set of proteins encoded by these genomes were common to all three genomes. In total, 61.8% of the genes were only present in two or one of the genomes. This was entirely unexpected. If three human genomes manifested such a small overlap in “core” genes, then we would not consider the genomes to be human. For that matter, such low gene content conservation might not exist in a comparison of three divergent vertebrate genomes. Suddenly, evolutionary biology had a new problem—extensive gene content variation in some prokaryotes. An explanandum without an explanans.

Research over the last 20 years has shown that most prokaryotes have pangenomes consisting of a core set of genes common to all members of the group and a set of accessory genes that are present in some, but not all members. Indeed, the complexity of the accessory component is further emphasized by its subdivision into shell and cloud components, the former referencing accessory genes with low-to-intermediate frequency in the dataset, while the latter referencing genes that are present in only one or a few genomes (Decano and Downing 2019). Pangenomes arise because of extensive horizontal gene transfer and gene loss (Tettelin et al. 2005; McInerney et al. 2017,  2020). In 2007, Hogg et al. (2007) analyzing 13 genomes of *Haemophilus influenzae* concluded that the largest class of genes in the accessory component of the pangenome (comprising 19% of the pangenome) are present only in a single genome. They further found that an average pair of genomes differs by around 300 genes. In fact, analyses of most prokaryotic pangenome datasets have identified a U-shaped distribution of gene frequencies—a large number of constant (core) genes, a sparse number of genes at intermediate frequency, and a large number of genes at low frequency in the dataset (Sela et al. 2021). This U-shaped distribution has been used to suggest that most accessory genes in a pangenome are transient, and consequently, accessory genes are most likely to be neutral. Furthermore, a direct correlation has been observed between a measure of “genome fluidity” and the effective population size of the species under study (Andreani et al. 2017; Vos and Eyre-Walker 2017). Larger pangenomes are found in species with larger effective population sizes and this has been used to argue that most accessory genes are neutral and are simply introduced because the populations are so large. Effective population sizes have been measured using direct measurements of mutation rates, or using d*N*/d*S* ratios, which is a proxy for the effectiveness of selection (Bobay and Ochman 2018).

The counterargument that I and others have made is that larger effective population size is associated with a greater efficiency of natural selection, which, in the absence of any other factor, would in fact remove variation, not cause it to increase (McInerney et al. 2017). Nonetheless, it is this efficiency of natural selection that is the major factor in pangenome origin and maintenance.

In organisms with enormous long-term effective population sizes, such as *E. coli*, we see selection overcomes random drift even where there are very small fitness effects, such as on synonymous polymorphisms at the third positions of codons (Sharp et al. 1993). Highly expressed genes often use a subset of codons for a specific amino acid that correspond to the most abundant tRNA molecules in the cytoplasm. While a mutation from one codon to a synonymous alternative does not change the encoded amino acid, there can still be a fitness effect because the change will either speed up or slow down the rate of translation, due to the difference in cytoplasmic abundance of the cognate tRNAs (Sharp et al. 1993). Organisms like *E. coli* have sufficiently large effective population sizes that the substitution of one silent nucleotide for another, in one amino acid position, in one protein in a genome encoding 5,000 proteins can be acted upon by natural selection. Using a data-driven approach, Wolf et al. (2016) estimated the percentage of genes in a pangenome that are effectively neutral to be 12%, while they estimated that 88% “evolve under a range of selective constraints”, while Sela et al. (2016) have been able to show that acquisition of genes is, on average, slightly beneficial. The conundrum of how organisms with such large effective population sizes (and associated efficiency of natural selection) can also manifest extensive accessory gene variation is resolved if we accept that the pangenome of any species with a large population size is also likely to be associated with several, perhaps thousands of niches and micro-niches (Domingo-Sananes and McInerney 2021). A selection-driven pangenome would then consist of genes whose fitness varies in different external environments.

Moving between external niches is only part of the story, however, because these niche-adapted genes might have positive or negative effects on other genes within the pangenome, and they in turn might have positive or negative effects on other genes, and so on, leading to an ecosystem-like set of relationships. This is the “evolving ecosystems” model of pangenomes. The combination of a fluctuating external ecosystem, with both biotic and abiotic drivers, combined with intra-genomic fluidity caused by gains and losses of genes, create the possibility that a gene contributes a multitude of different fitness effects to a pangenome. Indeed, changes in any given genome have the potential to abruptly shift the preferred ecological niche for the host, as is seen in, say *Campylobacter*, a common cause of human gastroenteritis, where the acquisition of three genes responsible for synthesizing vitamin B_5_ was shown to be important for colonizing cattle (Sheppard et al. 2013).

At this point, it is appropriate to acknowledge that a large number of mobile genetic elements exist, many of whom are selfish (Werren 2011). The dynamics of these mobile elements—frequently associated with genes that do not participate in mobility but are carried as passengers—has certainly played a major role in the evolution of the pangenomes we see today. In some respects, this is a consequence of natural selection, though the beneficiary is usually the selfish genetic element.

In a pangenome that is driven by selection, genes are conditionally beneficial or detrimental, or indeed they can vary in their essentiality (Beavan and McInerney 2022; Rosconi et al. 2022), and multiple niches exist into which the focal species can move, survive and thrive. The fitness effect of the presence or absence of a gene depends firstly on the external environment and whether or not the species can move between different niches, but also the fitness effect of a gene's presence or absence can depend on the content of the rest of the genome (Bapteste and Dupre 2013). It is interesting to note that intracellular parasites that live in a constant environment usually do not have extensive pangenomes or large effective population sizes (McInerney et al. 2017).

This resolution of the pangenome conundrum is also explicitly selectionist in the sense that it is explicitly non-neutralist. It is difficult to see that neutral mutation (in this case “mutation” simply means the acquisition of a new gene by horizontal gene transfer, or its loss during cellular replication) alone could give us the kinds of prokaryotic pangenomes that we see, particularly for those organisms with enormous effective population sizes. For a mutation to be truly neutral, it needs to have a fitness effect not substantially different to 1/*N_e_* and this could not change for the duration of the time it was polymorphic, which would be a large number of generations. The resolution we propose is also non-neutralist because we have evidence for at least some amount of selection governing the presence or the absence of some genes (Whelan et al. 2020, 2021; Hall et al. 2021). Adaptationist models have been quite rightly criticized in the past because of their dependence on “just so” stories (Gould and Lewontin 1979), but the counter argument must also apply—a neutral view of pangenome evolution must be accompanied by extensive evidence for the absence of selection. We can see evidence for neutral and nearly neutral evolution rather easily in silent substitutions in many eukaryotic genomes, particularly large eukaryotes (Doherty and McInerney 2013). In prokaryotes, the observation of “transient” genes at low frequency has been used as circumstantial evidence for the dominance of drift; however, it is equally plausible to suggest that complex internal and external interactions drive gene content variation.

We might be forgiven for thinking that there is a parallel between this discovery of such variation and the discovery in 1966 of unexpectedly high levels of protein polymorphism (Hubby and Lewontin 1966a, 1966b; Harris 1966, 1971). While it had been suspected for some time that diploid organisms harbored substantial levels of heterozygosity (Wallace 1958), from 1966 onwards starch gel electrophoresis could show quite definitively at the molecular level that human and other animal populations manifested amino acid replacement polymorphisms at many loci. Clearly, these new data needed to be interpreted in some kind of mechanistic way, and a debate ensued about whether these variations were selected or neutral (Harris 1971). In the end, the discovery of such variation led to the formalization of the Neutral Mutation—Random Drift Hypothesis (Kimura 1983) and the Nearly Neutral Hypothesis (Ohta 1973). One consequence of the interest in neutral mutations was the realization that in those organisms with large effective population sizes, the age of a neutral polymorphism, even one whose frequency is very low, was typically quite old—the expected length of time for a neutral allele to become fixed in a diploid population purely by drift is 4*N_e_* (Kimura and Ohta 1973). The parallel between now and then is to be found in the unanticipated diversity that is seen in many prokaryotic pangenomes, and this indeed mirrors the unanticipated polymorphism levels seen in proteins in the 1960s. The question today is whether all this diversity is selected, neutral or a combination of both (McInerney et al. 2017; Shapiro 2017; Vos and Eyre-Walker 2017).

So far, thousands of papers have produced valuable descriptions of pangenomes in terms of gene content, often accompanied by ad hoc identifications of likely adaptive gene acquisitions. Far less common has been the discussion of adaptive gene loss, though clearly it is important (Kuo and Ochman 2009, 2010). For example, there is evidence that loss of the *mspA* porin gene in *Mycobacterium tuberculosis* both enhances virulence and results in an increase in growth rate (Lamrabet et al. 2014). While the focus on describing pangenomes has been very useful, a more satisfying approach would interpret pangenomes in terms of gene- and system-level interactions within the pangenome, as well as an understanding of how the various genes within the pangenome interact with one another and with their immediate external environment.

Leaving aside issues about expression levels and appropriate promoters driving the expression of the gene, the fitness effect of an expressed gene depends on at least two features that are external to that gene. We know that the distribution of genes worldwide is highly nonrandom. The kinds of genes that can possibly be present is primarily selected by the local environment (Fondi et al. 2016). Next, the genetic background in which the gene finds itself also matters because this background determines the conflicts and cooperations a gene experiences (Hall et al. 2020). A naïve assumption might be that all genes in a genome might have a “unity of purpose”. That is to say, the mere existence of a gene is evidence enough that it works well with, and for, the rest of the genes in the genome, and that the totality of the gene complement helps to optimize the operations of the cell. In reality, a genome is successful if it can replicate, and produce more copies of itself. There is no reason to think that all the genes in a genome are united in the effort to achieve a particular aim, any more than we expect the presence of a bacterium in a pool of water to be sufficient evidence that the bacterium is there for the benefit of the other organisms in the pool.

Microbial communities are battlefields, with species vying with others for survival, while alliances and consortia constantly form and break up (Koenig et al. 2011). Prokaryotes live their lives under intense pressure from phage and protozoan predation. They can experience frequent ecological upheaval, and encounter competition for food and energy from both closely and distantly related organisms. This paradigm of constant change not only applies to microbes and their external environment, but also within the cell, where constant genomic change though time is a feature of all organisms. I argue here that continuously changing internal and external threats and opportunities create the conditions for the evolution of dynamic prokaryotic pangenomes that follow their own “ecological” rules. Specifically, I suggest the usefulness of looking at pangenome evolution through a lens that is normally associated with macroecology. In this treatment, the interacting and non-interacting ecological actors are genes within pangenomes, and not whole organisms living on a savannah, or in a swamp. Taking the perspective of the pangenome as an ecosystem (see table 1) allows us to re-frame the pangenome question in terms of different classes of interaction: competition (two interacting elements are harmed, resulting in decreased fitness for the cell), co-operation (both elements benefit), commensalism (one element benefits, the other is not impacted), and exploitation (one element benefits, the other is negatively impacted), to name a few. Pangenomes also are likely to contain many genes whose roles and functions are entirely orthogonal to one another. The analogy is not perfect but may act as a useful lens for further discovery in evolutionary biology.

**Table 1.** Hypotheses of Some Gene-to-gene Ecological Interactions that Can be Tested for Their Existence Within Pangenomes.

Gene-to-gene interaction	Sign	Effect	Example
*Mutualism, Reciprocal help*	+/+	Both genes benefit from interaction	Both genes collaborate in the same biochemical pathway.
*Commensalism*	+/0	One gene benefits, one is unaffected	*GeneB* produces a chemical useful to the cell, but it is also the precursor of a chemical produced by *GeneA* (therefore *GeneA* requires B but not *vice versa*).
*Competition*	−/−	Each gene affected negatively	Both carry out the same process (see e.g. (Bruns et al. 2018) ) and therefore only one copy can be in any given genome at the same time.
*Predation, Parasitism, Asymmetry*	+/−	One gene benefits, One is disadvantaged.	Selfish genetic elements that exploit the genome for self-propagation.

An ecological community is defined as a group of actually, or potentially interacting species living in the same place. The ecological community of the pangenome is the group of actually, or potentially interacting genes existing within the same pangenome. For operational reasons, we can describe this as a community that is bound together by a network of the influence that each gene (and of course its protein product) has on each of the other genes. We can think of different categories of effect that might occur when a gene is acquired by horizontal gene transfer or lost by deletion (Hall et al. 2020, 2021; Whelan et al. 2021). A direct effect would refer to the impact of the presence or absence of gene A on gene B in a two-gene interaction. An indirect effect would refer to the impact of the presence or the absence of gene A on gene C via an intermediary gene (A –> B –> C). A cascading effect is one that extends across three or more genes. A keystone gene is one whose presence or absence produces strong direct and indirect effects. It might be useful to explore a small set of examples.

Unless a gene is in a suitable genome, its fitness effect is likely to be deleterious. A hypothetical example might be a gene that is responsible for sugar transport being found in a methanogen genome. Methanogens are anaerobic and chemolithotrophic, with no interest in sugar transport. The possession by a methanogen of a sugar transporter gene would likely result in an additional burden to the methanogen of making the nucleotides for reproduction of this gene, and perhaps inadvertent transcription and translation of the gene would have additional metabolic costs. This is a “classic” situation—a gene is purged from a genome if its fitness effect on the host is sufficiently negative. This is natural selection in action, but it would not create a pangenome because the gene is most likely to be completely absent from the population. Conversely, the loss of a very useful gene encoding say, a ribosomal protein, is very likely to result in a large decrease in organismal fitness, meaning that its universal retention is also an example of selection in action, but this kind of selection would not result in a pangenome forming. Nonetheless, natural selection can result in the formation and maintenance of large pangenomes.

There are situations where a set of genes might be capable of contributing positively to the fitness of the host organism, but internal features of the genome can complicate the issue and result in the presence–absence variation. In the case of the bacterial genus *Salinispora*, we can see that there is a polymorphism that involves two biosynthetic gene clusters, both of which are responsible for the production of siderophores that facilitate iron acquisition for the cell. Both of these alternative siderophores are almost identical in function, though not in structure (Bruns et al. 2018). The more ancient desferrioxamine pathway has been replaced at least three times in the evolution of this genus by the alternative salinichelin pathway. Every genome in the genus has either one or other of these two clusters, but not both. Clearly, the two pathways are mutually exclusive, presumably because efficient bacterial genomes tend to be devoid of functional redundancy (Sorek et al. 2007). In this case, the function is adaptive and is “core” to this group of organisms and the presence of at least one of the biosynthetic gene clusters is selected by the external environment, but the genes compete with one another. Exchange of one biosynthetic cluster for another is likely to be a neutral substitution, having no effect on the overall fitness of the host, but retention of the function is strongly selected. The presence or the absence of these two clusters mean they are part of the overall accessory genome of *Salinispora*. Other examples of when competition could contribute to presence–absence variation would include plasmid incompatibility (Novick 1987), and the competition for Transcription Factors by multi-copy genes (Brewster et al. 2014).

Persistent polymorphism is observed in pangenomes, where genes are under selection that prevents them from ever becoming part of the core genome. There are several possible explanations for polymorphism maintenance. For instance, Negative Frequency-Dependent Selection which was developed initially as a mathematical model (Wright 1948), but later empirically shown in snails (Clarke and O’Donald 1964), has been reported as an explanation for changes in gene frequency in multi-strain populations (MSPs) of *Streptococcus pneumoniae* (Harrow et al. 2021), as well as persistent polymorphism in vaccine-associated gene frequency dynamics (Corander et al. 2017). In a different situation, Morris et al identified what they called the “Black Queen Hypothesis” where essential genes could be lost from individual genomes, though retained in the pangenome through the maintenance of collaborative functions (Morris et al. 2012). This shows that pangenomes respond to external stimuli and the eco-evo perspective is necessary to explain gene content variation.

For some time, we have been interested in exploring “surprising co-incidences” in pangenomes, which could be indicative of mutualism or co-operation (always with the caveat that correlation does not equal causation). We have developed the CoinFinder tool (Whelan et al. 2020) for this purpose. The approach seeks to identify pairs of genes that co-occur more often than if they were randomly distributed throughout the focal clade (with appropriate correction for multiple testing). Additionally, we pay particular attention to sets of genes that have a phylogenetic distribution pattern that does not correspond to the backbone phylogeny of the group of interest—in other words, we identify genes that have tended to be inherited together through HGT events. By looking for these kinds of pairs of genes, we are explicitly looking for genes whose co-occurrence might be best explained by mutualistic benefits, synergy, or dependence. We represent these significant co-occurrences as a network, with each gene family being represented as a node and each statement of significant co-occurrence being denoted by an edge connecting two nodes. Each gene family is of course, a priori, free to co-occur with any other, but when we have examined real datasets, we find a highly structured set of co-occurrence relationships. To demonstrate that this “ecological” approach had potential, we focused on the V-ATPase in 534 *Streptococcus pneumoniae* genomes (Whelan et al. 2020). V-ATPase is a multi-protein enzyme whose primary role in *S. pneumoniae* is to create a proton gradient using energy from ATP hydrolysis, to maintain the intracellular pH via proton extrusion. The enzyme is expected to consist of 9 proteins. Standard annotation of *S. pneumoniae* genomes only identified 6 proteins, but CoinFinder identified another three that always co-occurred with these proteins. On further analysis, we could say with reasonable confidence that these were the “missing three” proteins of the complex. Intriguingly, another 51 genes, encoding neuraminidases, genes involved in DNA transformation, ISS6-related proteins and genes implicated in virulence, among others, also showed a co-occurrence pattern with some or all the V-ATPase genes. This example again shows the benefit of using an ecosystem approach: we tested whether a signature of mutualism/synergy/dependence was present in the *S. pneumoniae* pangenome, we then used the results to hypothesize that unnamed members of the “ecosystem” were really elements of the V-ATPase complex, we have further proposed that 51 other actors are “of interest” with respect to the complete understanding of V-ATPase evolution in *S. pneumoniae*.

##  

### Future Perspective

We remain almost completely in the dark about the complexity of the interactions between the genetic elements in pangenomes, the fitness effects of acquired and lost genes, and the overall theory by which pangenomes are governed. It is arguable that in our search for the match between an organism and its environment that we have prioritized the link between genes and ecosystems and have not focused as much on the interactions between genes. Given that we see pangenomes in most prokaryote species, not fully understanding the effects that genes in a genome have on one another is an important gap in our knowledge.

We might also ask: “*Cui bono*?” Who benefits from the pangenome? Traditionally, the advantages or disadvantages of genetic change are described in terms of the effect on the host organism and usually the outcome is the extent to which the change enhances or diminishes the likelihood of passing their genes on to the next generation. Clearly, selfish genetic elements and addiction systems present a problem for that kind of perspective. Additionally, if a useful genetic construct is assembled in one organism and is subsequently horizontally transferred to another, then we cannot really say that the ultimate beneficiary is the species in which the construct originated. Given the prevalence of HGT, can we ever say that the benefit of pangenomes is ever to the organism, and not to the genes themselves?

A good theory base would ideally possess all seven of the desiderata that Bunge (Bunge 1987) has identified for rationality. Therefore, we need to address 1) the *conceptual basis* of pangenomes, thereby reducing fuzziness in the concept, 2) we need a *logical* (consistent) theory, 3) we wish it to be *evidence-based*, 4) we care for the *epistemological* basis of the theory and the routes to knowledge, 5) we wish to have a *rational ontology*, consistent with scientific theory, 6) we wish to pursue this goal because of its *worth*, and 7) we wish to develop *practical* approaches to further our understanding.

It is quite true that the conceptual basis for pangenomes remains fuzzy. When the first genome of a species is sequenced, then the “core” is 100% of the genes. As each subsequent isolate’s genome is sequenced, the core is likely to reduce and the number of genes in the accessory component might increase. This means that the pangenome might be operationally defined by the current sampling of any given species or group, but this is not a concept, it is a convenience. Additionally, it is common to see the accessory genome being described as a dispensable component, but there is evidence that some genes whose presence is variable across the dataset, are not dispensable either. Conversely, some genes might be found in all genomes of a sample and are not essential or are conditionally essential (Beavan and McInerney 2022; Rosconi et al. 2022). A pangenome concept needs to be independent of sampling.

If theory is the over-arching description of the mechanism through which pangenomes arise and are maintained, then I feel that the broad outlines are emerging, though feedback from data analysis will be necessary to gain a complete understanding. Methods continue to be developed, the influences of genes on one another, combined with the influence of the external ecosystem on the internal genomes are being investigated with rigor. The beginning of a rational ontology for pangenomes began, naturally, with the discovery of extensive gene gain and loss in organisms that were otherwise considered to be close relatives, several decades after the first genetic investigations of prokaryotes. However, a rational ontology currently feels incomplete. Control of genes, feedback and feedforward loops, defence and attack mechanisms, and swapping of promoter regions are all understudied in the context of the pangenome and it is likely that there is a wealth of unknown features that are inherent to the pangenome’s existence. There might be features that we do not study simply because we do not know of their existence in the first place.

I argue that we have not yet achieved all Bunge’s desiderata, and that a multidisciplinary approached, combined with the proposed ecological perspective can facilitate an outcome where all seven desiderata are satisfied.
